# External quality assessment for acid fast bacilli smear microscopy in eastern part of Ethiopia

**DOI:** 10.1186/s13104-015-1478-0

**Published:** 2015-10-05

**Authors:** Desalegn Admassu Ayana, Zelalem Teklemariam Kidanemariam, Habtamu Mitiku Tesfaye, Fitsum Weldegebreal Milashu

**Affiliations:** Department of Medical Laboratory Science, College of Health and Medical Sciences, Haramaya University, East Harerge, Ethiopia

**Keywords:** EQA, Sputum smear microscopy, Blinded rechecking, Onsite evaluation, Panel test

## Abstract

**Background:**

External quality assessment (EQA) of sputum smear microscopy is essential and indispensable component of any tuberculosis program. This study assessed the EQA of acid fast bacilli (AFB) smear microscopy through onsite evaluation, blinded rechecking and panel test. A one year study was conducted on eight health institution laboratories from December 2011 to December 2012. Onsite evaluation, blinded rechecking and panel tests were used to collect data. Data were analyzed using SPSS version 16. Sensitivity, specificity, predictive values, and proportions of false readings were calculated. The level of agreement was measured using Kappa (κ) value.

**Results:**

Problems observed during onsite evaluation include shortages of materials, disinfectant, and poor storage and working condition. A total of 578 slides were collected for blinded rechecking, of which 102 (17.6 %) were reported as positive by peripheral laboratories. The panel test revealed an overall error of 17 (25.25 %) of which 14 (17.5 %) were minor errors [low false negative 6 (7.5 %) and low false positive 8 (10 %)], and 3 (3.75 %) were major errors (high false positive). The sensitivity, specificity, positive predictive values (PPV) and negative predictive values (NPV) of the peripheral laboratories were 83.5, 97.8, 91.7, and 95.7, respectively. The false readings at the peripheral laboratories were 32 (5.5 %). Agreement on reading the slides was observed on 546 (94.5 %) slides (K = 0.84, SE = 0.054).

**Conclusions:**

Lack of reagents, supplies, favorable working environment and AFB related technical problems were identified in the peripheral laboratories. High false negative error was found to be the predominant major error. A continuous and strong EQA scheme should be implemented to avoid reporting errors and produce quality sputum results.

## Back ground

The annual estimated incident cases of Tuberculosis (TB) were 300,000 of which 46 % were smear positive [1].

There have been changes in laboratory diagnostic techniques that have been used for the diagnosis of pulmonary tuberculosis. Direct sputum smear microscopy is the most cost effective tool for diagnosing patients with infectious TB and for monitoring their progress on treatment [2–4]. The sensitivity of the test has been reported to be variable ranging from 20 to 60 %. The proportion is lower among patients co-infected with human immunodeficiency virus (HIV) and children [4–6]. The lack of time and laboratory expert to make thorough searches of each field under the microscope is in part related to the low sensitivity of this method [1].

Errors in sputum smear reading may result in failure to detect persons with infectious TB, undetected patients can then continue to spread infection in the community, or unnecessary treatment. External quality assessment (EQA) of acid fast bacilli (AFB) sputum smear microscopy is a process that assesses peripheral laboratory performance by higher-level laboratory and it includes blinded rechecking, on site evaluation and panel testing [3]. Quality assessment is essential to determine the source of performance problems and take remedial actions [1].

Most literatures focused on blinded rechecking of stained slides even though on site evaluation and panel testing are known to be valuable and costly. A blinded rechecking of slides in Taiwan revealed that 2 out of 4 laboratories had at least 1 high false negative (HFN) and 3 out of 4 laboratories had at least 1 low false negative (LFN) result [7]. A similar blinded rechecking study conducted in 12 microscopy centre laboratory technicians in India had found a false reading result that range from 2 to 7 % between participating laboratories [8]. A study conducted in Burundi has reported false positive results of as high as 6.9 % and a false-negativity of 3.4 % [9]. In Southern part of Ethiopia, Shargie and his colleagues conducted a blinded rechecking study that showed an overall false reading of 3.2 % and an overall agreement of 96.8 % [10]. According to the Association of Public Health Laboratories/Centers for Disease Control and Prevention (APHL/CDC) guideline, reliability in double-blind readings of sputum smear microscopies is expected to be near 95 % for highly positive smears (AFB++ and AFB+++), and from 30 to 50 % for inconclusive smears (1–9 AFB/100 fields) [3].

This study applied all the EQA standards in order to give a far reaching insight into the quality of sputum smear examination. There was no previous report on the status of laboratory quality performance of health facilities from Eastern part of Ethiopia, particularly on sputum smear examination using Ziehl–Neelsen (ZN) staining techniques. Therefore, this study assessed the EQA of AFB smear microscopy using ZN staining in Eastern part of Ethiopia following the three standards of EQA to give the real practice of sputum smear microscopy.

## Methods

### Study setting

A 1 year study was conducted from December 2011 to December 2012 on laboratories that have been practical attachment sites for students of the College of Health and Medical Sciences, Haramaya University. The study laboratories were include from Dire Dawa Administrative council, Harari regional state and Eastern Hararghe zone of Oromiya Regional state. These regions and administrative council are found about 500–570 km away from the capital city, Addis Ababa. The study laboratories include Dilchora hospital in Dire Dawa and Jugel hospital in Harar, and six health center laboratories (Gursum, Haramaya, Babile, Lege Hare, Melka Jebdu and Sabian).

### Data collection instrument and procedures

Data were collected using on site evaluation checklist, blinded rechecking and panel tests following EQA for AFB smear microscopy guideline [3]. All peripheral laboratories included in the study have employed ZN staining technique for detection of AFB in a sputum smear. In short, sputum samples were collected, smeared, air dried and fixed. The staining was performed using Carbol fuchsin, acid alcohol and methylene blue. The stained slides were dried and examined using 100× objective and positive slides were graded based on the bacilli number observed [11]. All stained slides were collected from peripheral laboratories for blinded rechecking and unstained panel test slides were stained by ZN Technique, examined and graded using 100× objective in all study laboratories following the standard procedure.

### On site evaluation

This was conducted using the standard checklist [3] to assess the different aspect of working environment inside TB laboratory.

### Blinded rechecking

All slides reported as ‘Positive for AFB’ and ‘No AFB seen’ using ZN staining technique during the study period were collected from peripheral laboratories. The number of slides to be collected from each study laboratory was determined based on the number of smear positive slides seen in the same quarter according to sample size calculation criteria on EQA for AFB smear microscopy guideline [3].

In brief, the peripheral laboratories were informed to retain all positive and negative ZN stained slides with their results filled in a separate form. The slides were collected prospectively every quarter (four times during the study period), transported to and examined in Haramaya University laboratory. The study sites and the collected slides were coded and read using 100× objective by senior Medical Laboratory Technologist (second reader) from Medical Laboratory Science department, Haramaya University. Slide reading results from peripheral laboratories were kept confidential from the second reader. Discrepant readings between the peripheral laboratory reading and the second reader were re-read and verified by a third senior Medical Laboratory Technologist from the Medical Laboratory Science department, Haramaya University.

### Panel testing

For the panel testing, positive and negative sputum samples were collected and processed in Haramaya University laboratory. Negative smears and different grades of positive smears were prepared for panel testing. Samples were taken from each negative and positive prepared slides and were stained by ZN staining technique to check for quality of smears and grades of positive slides using 100× objective. Unstained panel test slides were then dispatched to the peripheral laboratories. A total of 80 panel test slides with different results were prepared and 10 slides (five negative, two 1–9, one 1+, one 2+ and one 3+) were packed and sent to each peripheral laboratory as per the acceptable slide set with increasing degree of difficulty. The results were analyzed based on the determination of acceptable performance (passing score) using one of the scoring systems proposed (i.e. using set of 10 slides, each slide is worth 10 points, total possible score = 100, any positive called negative scores 0, any negative called positive scores 0, quantification error [2 grades) scores 5 and passing score = 80]. All the laboratory procedures were conducted following the standard operating procedure (SOP) [3].

### Data management and analysis

The quality of data was checked by reviewing the questionnaire for coherence and completeness. Data were entered, cleaned and analyzed using SPSS version 16. Results were presented as percentages in tables, and Chi square tests were used to assess the difference in blinded rechecking results between the peripheral and the final reading results. p value less than 0.05 at 95 % confidence interval was considered statistically significant. The sensitivity, specificity, positive predictive values (PPV) and negative predictive values (NPV) of the peripheral diagnostic laboratories were calculated by considering the final reading/re-reading result as the gold standard for the true smear result. The level of agreement between the peripheral laboratory and University laboratory was measured using Kappa (κ) value. Major and minor errors were calculated. Major errors indicate gross technical deficiencies, and include both high false positive (HFP) and high false negative (HFN) errors. It was considered as HFP when a negative smear was misread as 1+ to 3+ and HFN when a 1+ to 3+ positive smear was misread as negative. Minor errors indicate low false positive (LFP), low false negative (LFN) and quantification error (QE). It was considered as LFP when a negative smear was misread as low positive (1–9AFB/100fields) and LFN when a low positive smear (1–9AFB/100fields) was misread as negative. QE is difference of more than one grade in reading a positive slide between examinee and controller [3].

### Ethical consideration

Ethical clearance was obtained from the Institutional Health Research and Ethical Review Committee of Haramaya University. All the laboratories and people included in the study agreed to participate in the study through informed consent. All information obtained from participating laboratories during the study was kept confidential.

## Results

### On-site evaluation

A total of 33 Medical Laboratory professionals were working in the institutions, of which 18 were Diploma and 15 were Bachelor of Science (B.Sc) graduates in the field of Medical Laboratory Technology during the study period. All the laboratories had claimed to have SOP for AFB smear microscopy using ZN technique. Seven of the laboratories had reported that their staff members had participated in different trainings within the last 2 years. Three sputum specimens were routinely collected by all peripheral laboratories. All the laboratories had reported to have a staff profile change in number (high turnover) and academic status (upgrade from diploma to Bachelor of Science degree level) within the last 1 year. Shortage of supplies such as distilled water, lens tissue, disinfectant, laboratory chemicals and reagents, and poor storage condition were among the problems identified in the peripheral laboratories. Besides, all laboratories didn’t have an analytical balance and a separate area for TB work. And large numbers (87 %) of peripheral laboratories did not use personal protective equipment (PPE) and appropriate cleaning procedure. Moreover, 87.5 % of peripheral laboratories did not clean their microscope objectives after reading positive slide and they did not have daily maintenance, 87.5 % did not filtered reagents, 62.5 % of them did not prepare slides with appropriate thickness, 37.5 % had unacceptable background staining, and 25 % had never used AFB quality control; only 50 % allocated sufficient slide reading time (more than 10 min for examination of 100 fields of a slide), but 50 % of them had high work load and poor laboratory data management. Even though, 87.5 % claimed to have taken part on EQA scheme, only three of them received feedback on their performance from the central EQA office [Ethiopian Health and Nutrition Research Institute (EHNRI)] (Table 1).

**Table 1:** Most common problems reported or observed during on-site evaluation visits of the eight selected health institution medical laboratories from December 2011 to December 2012

Problems found	Laboratories N (%)
Laboratory safety	
No separate area for TB work	8 (100)
No adequate ventilation	5 (62.5)
No biohazard waste bin with a lid	4 (50)
Improper use of PPE	7 (87.5)
Inappropriate cleaning procedure	7 (87.5)
Laboratory reagents	
Expired reagents or no label	3 (37.5)
Not filtered before use	7 (87.5)
Filtered once a month	4 (50)
Laboratory supplies	
No staining rack	5 (62.5)
No wire loop	6 (75)
No funnel	4 (50)
No analytical balance	8 (100)
Microscope	
Not sufficient	4 (50)
Inadequate light source	6 (75)
Objective not cleaned after positive slide	7 (87.5)
No routine care/daily maintenance	7 (87.5)
Smearing and staining procedures	
Background staining not acceptable	3 (37.5)
Background material doesn’t represent sputum	4 (50)
Inappropriate smear thickness	5 (62.5)
Inappropriate smear size	4 (50)
Report with grading	6 (75)
Include control smears	
Daily	2 (25)
New batch of reagents	5 (62.5)
Never	2 (25)
Time taken to examine 100 fields	
5 min	4 (50)
10 min	3 (37.5)
15 min	1 (12.5)
Administrative	
High workload	4 (50)
Incomplete laboratory request form	7 (87.5)
No NTP approved report forms	3 (37.5)
EQA	
Participated	7 (87.5)
Rechecking result received	3 (37.5)

### Blinded rechecking

A total of 578 slides were collected from eight hospital and health center laboratories. An average of 24 slides (ranging from 22 to 28) were collected from each peripheral laboratory following the standard procedure. And about 102 (17.6 %) of the collected slides were reported positive and 476 (82.4 %) were negative at the peripheral laboratories (first reader) (Table 2).

**Table 2:** Agreement in readings of slides among selected health institution laboratories and final rereading by a third reader from December 2011 to December 2012

Peripheral laboratory	Final re reading			
	Positive	Negative	Total	
Positive	90	12	102	χ^2^ = 3.87 P = 0.00
Negative	20	456	476	
Total slides	110	468	578	

A total of 32 (5.5 %) discrepant slide results were obtained between the peripheral laboratory and Haramaya University laboratory reading (second reader). Discrepant results between peripheral and Haramaya University laboratory were further re-read by another laboratory technologist from Haramaya University (third reader) and yield similar result with the second reader. From the 32 discrepant slides, major errors (HFN and HFP) were found in 23 (4 %) of the slides, of which 20 (3.5 %) of the slides were HFN and 3 (0.5 %) were HFP. The HFN error was observed in 5 (62.5 %) of the peripheral laboratories. Minor errors, such as LFP and Quantification Errors (QEs), were observed in all of the peripheral laboratories. The sensitivity, specificity, PPV and NPV of the peripheral laboratories were on average 83.5 (50–100 %), 97.8 (82.6–100 %), 91.7 (33.3–100 %), and 95.7 (87–100 %), respectively. The agreement between the peripheral laboratories and Haramaya University laboratory reading was 94.5 % (K = 0.84, SE = 0.054) (Table 3).

**Table 3:** Sensitivity, specificity, PPV and NPV of sputum microscopy of the selected laboratories from December 2011 to December 2012

Laboratories coded	Sensitivity (%)	Specificity (%)	PPV (%)	NPV (%)	Kappa value
1	85	100	100	94	0.89
2	100	100	100	100	1
3	75	100	100	95.2	0.83
4	50	100	100	87	0.74
5	100	100	100	100	1
6	100	100	100	100	1
7	90.9	100	100	94.4	0.92
8	66.7	82.6	33.3	95	0.34
Average	83.54	97.82	91.7	95.75	0.84

### Panel tests

From the total of 80 panel test slides (10 slides for each site), the overall quantification error was 37 (46.25 %) and the average panel scoring result was 78.25 % (Table 4). The overall error was 17 (25.25 %) of which minor errors were 14 (17.5 %) (i.e. 6 (7.5 %) LFN and 8 (10 %) LFP), and major errors were 3 (3.75 %) (i.e. 3 (3.75 %) HFP) (Table 5).

**Table 4:** Panel test results of the selected health institution laboratories from December 2011 to December 2012

Laboratories studied coded	Number of slides stained and read	% of discordant result					
		Negative (n = 5 slides)	1–9 AFB/100 (n = 2 slides)	+1 (n = 1 slide)	+2 (n = 1 slide)	+3 (n = 1 slide)	Total
		No. (%)	No. (%)	No. (%)	No. (%)	No. (%)	No. (%)
1	10	0 (0)	1 (50)	0 (0)	0 (0)	0 (0)	1 (10)
2	10	2 (40)	1 (50)	1 (100)	0 (0)	1 (100)	5 (50)
3	10	1 (20)	2 (100)	0 (0)	1 (100)	1 (100)	5 (50)
4	10	2 (40)	2 (100)	1 (100)	1 (100)	0 (0)	6 (60)
5	10	2 (40)	2 (100)	1 (100)	1 (100)	1 (100)	7 (70)
6	10	2 (40)	1 (50)	0 (0)	0 (0)	1 (100)	4 (40)
7	10	1 (20)	1 (50)	1 (100)	0 (0)	1 (100)	4 (40)
8	10	1 (20)	2 (100)	0 (0)	1 (100)	1 (100)	5 (50)

**Table 5:** Classification errors of AFB panel test slides reading status of laboratory professionals among the peripheral laboratories from December 2011 to December 2012

Panel slides		Number of slides read	Error result: N (%)					
			QE	LFN	LFP	HFP	HFN	Total errors
Un stained slides	No AFB	40	0 (0)	0 (0)	8 (20)	3 (7.5)	0 (0)	11
	AFB positive	40	0 (0)	6 (15)	0 (0)	0 (0)	0 (0)	6 (15)
	Total	80	0 (0)	6 (7.5)	8 (10)	3 (3.75)	0 (0)	17 (25.25)

*HFN* high false negative, *HFP* high false positive, *QE* quantification error, *LFN* low false negative, *LFP* low false positive

## Discussion

The onsite evaluation result had shown that all the laboratories included in the study didn’t have a separate area for TB work. Most peripheral laboratories reported that they did not run control at the time of opening new batches of reagent. In nearly one-third of the stained slides collected for blinded rechecking, the background didn’t represent sputum. The absence of separate area for TB work may create unfavorable working environment which may result in poor sample collection and slide preparation techniques for the professionals which may, in turn, affect the quality of test results. In this study, most peripheral laboratories were reported EQA participation, but less than 50 % of them were received feedback on their performance. This may be due to the distance and the high work load of the central reference laboratories (EHNRI) to regularly conduct PTs, collect results and send feedback. Among the EQA standards, on-Site field visit is preferred to obtain a realistic assessment of the conditions and skills practiced in the laboratory. The major problems in Pulmonary TB diagnosis were reported to be low detection of smear-positive and over-diagnosis of smear-negative slides [3]. This study found an overall false reading of 5.5 % between the peripheral laboratory and Haramaya University laboratory during the blind rechecking of stained slides. This result falls within the false reading result range of 2–7 % which was reported by a study conducted in India between participating laboratories [8]. Compared to a study conducted in the southern part of Ethiopia, our finding, 3.2 %, was relatively higher in an overall false reading. The difference might be attributed to the problems reported during the onsite evaluation, working environment and the difference in the level of training of laboratory staff in the peripheral laboratories studied. Complaints and dissatisfaction reflected by the professionals may result in poor laboratory results [10]. Blinded rechecking can facilitate assessment of laboratory professionals at peripheral laboratory. But the inconsistency or absence of strong EQA scheme may also contribute to the problem [12].

From the 5.5 % discrepant slides reported in the blinded rechecking, 4 % were major errors of which, 3.5 % were reported as HFN and 0.5 % were HFP. This study found similar false negative and much lower false positive results compared to the study conducted by Buzingo and his colleagues (false negative of 3.4 % and false positive as high as 6.9 %) [9]. The variation in false positive result may be due to the difference in study settings, work environment and training level of the laboratory professionals. A false-positive result can cause severe consequences besides incurring financial costs, and a false-negative result causes harm to the patient due to the delay in diagnosis, incurs costs on society, and brings about loss of faith in the services offered by the laboratory [3, 4, 9, 13]. Therefore, laboratories should undergo extensive review of their procedures and participate in a slide rechecking program in order to produce quality results.

In this study, blinded rechecking had found an overall agreement of 94.5 %. According to the APHL/CDC guideline, reliability in double-blind readings of sputum smear microscopies is expected to be near 95 % for highly positive smears (AFB++ and AFB+++). Our result was in line with the one set by the WHO. Although the distinction indicated by the plus signs is not an essential condition for the diagnosis of tuberculosis, it is important for the treatment follow-up to effectiveness of the medications prescribed by health professionals [3, 14].

An irregular report is inevitable in AFB microscopic examination, as it is difficult to attain a uniform distribution of organisms on the slide. Likewise, AFB is not homogeneously distributed in sputum and very few may be detected in an examination of 100 fields by one technician. Thus, different laboratory professionals examining 100 fields of a slide might not get similar results, [3, 15].

Panel testing is conducted to determine whether laboratory professionals can adequately perform AFB smear microscopy. This method evaluates individual performance in staining, reading and reporting of AFB slides [2, 9].

The overall error in the panel test was more than 25 %. The average panel scoring result (78.25 %) of this study was close to the acceptable performance (passing score) of panel tests which is (80 %) [3].

The limitations of this study include the difficulty to avoid slide selection bias during selection of slides for rechecking as laboratory professionals at peripheral laboratories may tend to retain slides with good quality staining regardless of the instruction before data collection.

## Conclusions

Lack of reagents, supplies, favorable working environment and AFB related technical problems were identified in the peripheral laboratories in this study. High false negative error was found to be the predominant major error. A continuous and strong EQA schemes should be implemented at each laboratory to avoid reporting errors and produce quality sputum results. Engaging higher education institutions in a regular EQA scheme in their respective areas may support to improve the quality of laboratory results.

## Abbreviations

AFB: acid fast bacilli
APHL/CDC: Association of Public Health Laboratories/Center for Disease Control and Prevention
DOTS: directly observed treatment strategy
EQA: external quality assessment
IUATLD: International Union against Tuberculosis and Lung Disease
NTP: National tuberculosis Program
SOP: standard operating procedure
